# Variants of the Sir4 Coiled-Coil Domain Improve Binding to Sir3 for Heterochromatin Formation in *Saccharomyces cerevisiae*

**DOI:** 10.1534/g3.116.037739

**Published:** 2017-02-10

**Authors:** Anke Samel, Adam Rudner, Ann E. Ehrenhofer-Murray

**Affiliations:** * Institut für Biologie, Humboldt-Universität zu Berlin, Berlin 10099, Germany; † Department of Biochemistry, Microbiology and Immunology, Ottawa Institute of Systems Biology, University of Ottawa, Ontario ON K1H 8M5, Canada

**Keywords:** gene silencing, repression, chromatin, Sir1

## Abstract

Heterochromatin formation in the yeast *Saccharomyces cerevisiae* is characterized by the assembly of the Silent Information Regulator (SIR) complex, which consists of the histone deacetylase Sir2 and the structural components Sir3 and Sir4, and binds to unmodified nucleosomes to provide gene silencing. Sir3 contains an AAA^+^ ATPase-like domain, and mutations in an exposed loop on the surface of this domain abrogate Sir3 silencing function *in vivo*, as well *in vitro* binding to the Sir2/Sir4 subcomplex. Here, we found that the removal of a single methyl group in the C-terminal coiled-coil domain (mutation T1314S) of Sir4 was sufficient to restore silencing at the silent mating-type loci *HMR* and *HML* to a Sir3 version with a mutation in this loop. Restoration of telomeric silencing required further mutations of Sir4 (E1310V and K1325R). Significantly, these mutations in Sir4 restored *in vitro* complex formation between Sir3 and the Sir4 coiled-coil, indicating that the improved affinity between Sir3 and Sir4 is responsible for the restoration of silencing. Altogether, these observations highlight remarkable properties of selected amino-acid changes at the Sir3-Sir4 interface that modulate the affinity of the two proteins.

The genome of eukaryotic organisms is packaged with histone and nonhistone proteins into chromatin, which is the substrate for all processes on the genetic material, like DNA replication, transcription, DNA repair, and chromosome segregation. The chromatin architecture differs among different genomic regions, and allows the organism to implement individual gene expression programs according to cellular function, for instance, during development (Ehrenhofer-Murray 2004). Large regions assume a repressive structure termed heterochromatin, which is thought to result from a more condensed folding of the chromatin fiber, and is brought about by heterochromatin proteins that bind to the nucleosomes. In higher eukaryotes, such regions typically are found at the telomeres, where they prevent degradation and recombination, and centromeres, where they are important for proper chromosome segregation (Perrod and Gasser 2003).

An archetypal form of heterochromatin is found in the budding yeast *Saccharomyces cerevisiae* at the silent mating-type loci *HML* and *HMR* and at the telomeres (Rusche *et al.* 2003). The establishment and formation of heterochromatin at these loci is mediated by the silent information regulator (SIR) complex, which consists of the NAD^+^-dependent histone deacetylase (HDAC) Sir2, and the structural components Sir3 and Sir4 (Kueng *et al.* 2013). All three components of the complex are necessary for transcriptional gene silencing (Rine and Herskowitz 1987). The establishment and spreading of silent chromatin is a stepwise process, in which the SIR complex does not directly bind to the DNA, but is recruited via sequence-specific DNA binding proteins, and undergoes specific contacts with the histones in the nucleosomes (Oppikofer *et al.* 2013a). In a first step, a Sir2/Sir4 subcomplex binds to proteins/protein complexes like Rap1 (Moretti *et al.* 1994), the origin recognition complex (ORC) (Triolo and Sternglanz 1996), and Abf1, which themselves bind *cis*-acting DNA sequences (Kimmerly *et al.* 1988), the so-called silencer elements. At the *HM* silencers, this interaction is bridged by the Sir1 protein (Bose *et al.* 2004). Subsequently, Sir2 deacetylates the lysines in the N-termini of histones H3 and H4, including H4 lysine 16, which is essential for effective silencing (Imai *et al.* 2000; Landry *et al.* 2000). This leads to the recruitment of Sir3 to the unmodified nucleosomes (Hecht *et al.* 1995). This process of deacetylation of histones and SIR protein binding is repeated in multiple cycles, and allows the SIR complex to spread along the chromatin fiber (Rusche *et al.* 2002). The extent of SIR spreading depends on the concentration of each component, as well as on histone acetylation as the substrate for Sir2 deacetylation, and overexpression of Sir3 leads to extension of the silent region (Renauld *et al.* 1993; Maillet *et al.* 1996).

A mechanistic understanding of heterochromatin architecture requires detailed molecular insights into the interactions among the SIR proteins. Structural information is available for several homologs of Sir2 (Marmorstein 2004), as well as for *S. cerevisiae* Sir2 bound to a fragment of Sir4 (Sir2 interaction domain SID, aa 737–893) (Hsu *et al.* 2013). Sir2 consists of a Rossman fold domain, and a smaller zinc-containing regulatory domain, and the Sir4 fragment contacts the interface between the N-terminal regulatory domain, and the catalytic domain. Sir2 forms a stable heterodimer with Sir4, and this interaction strongly stimulates the HDAC activity of Sir2 (Tanny *et al.* 2004; Cubizolles *et al.* 2006; Hsu *et al.* 2013).

Sir3 shares its domain structure with its paralog Orc1, a component of the replication initiation complex ORC (Norris and Boeke 2010). It consists of three functional domains: the N-terminal bromo-adjacent homology (BAH) domain, the AAA^+^ ATPase-like domain, and the C-terminal winged helix-turn-helix (wH) domain (Figure 1A). (1) The BAH domain (aa 1–214) is a nucleosome-binding module (Onishi *et al.* 2007; Sampath *et al.* 2009), whose structure with the nucleosome shows important contacts with at least 28 histones residues, and whose binding is inhibited by acetylation of H4 K16 and methylation of H3 K79 (Armache *et al.* 2011; Wang *et al.* 2013), but enhanced by Nα-acetylation of Sir3 (Arnaudo *et al.* 2013; Yang *et al.* 2013). (2) The C-terminal 138 amino acids (aa) of Sir3 show a variant winged helix-turn-helix conformation that forms a dimer, and dimerization is essential for its silencing capacity. This domain also contributes to nucleosome binding of the SIR complex, but does not itself bind to chromatin (Oppikofer *et al.* 2013b). (3) Our structural analysis of the Sir3 AAA^+^ domain (Ehrentraut *et al.* 2011) revealed the typical structures of a base and a lid subdomain, as observed in other AAA^+^ ATPases, but an unusual topology of the domains relative to each other that disfavors nucleotide binding in the cleft between the domains. In contrast to other AAA^+^ ATPases, Sir3 lacks the residues required for ATP binding and hydrolysis (Bell *et al.* 1995). We identified several mutations in the Sir3 AAA^+^ domain that abrogate Sir3 silencing function (Ehrentraut *et al.* 2011). In particular, mutations in an extended loop of the base subdomain that connects α-helix 4 to strand 3 of the central β-sheet (K657A, K658A, and allele *sir3-1067*) cause a strong loss of Sir3 silencing function, and abrogates the *in vitro* interaction of Sir3 with Sir2/Sir4. Conversely, mutations in a loop connecting the α-helices 3 and 4 (D640A, S642L, and allele *sir3-1021*) abrogate *HM* silencing, but do not affect the Sir3–Sir4 interaction. The AAA^+^ domain is also able to bind nucleosomes *in vitro* in a H3 K79 methylation-sensitive fashion, and thus also contributes to chromatin binding of full-length Sir3. In addition to these three domains, structural information is also available for a short fragment of Sir3 that interacts with Rap1. This region (aa 456–483) lies N-terminal to the AAA+ domain, and forms a short α-helix that binds to a C-terminal region of Rap1 (Chen *et al.* 2011).

**Figure 1:**
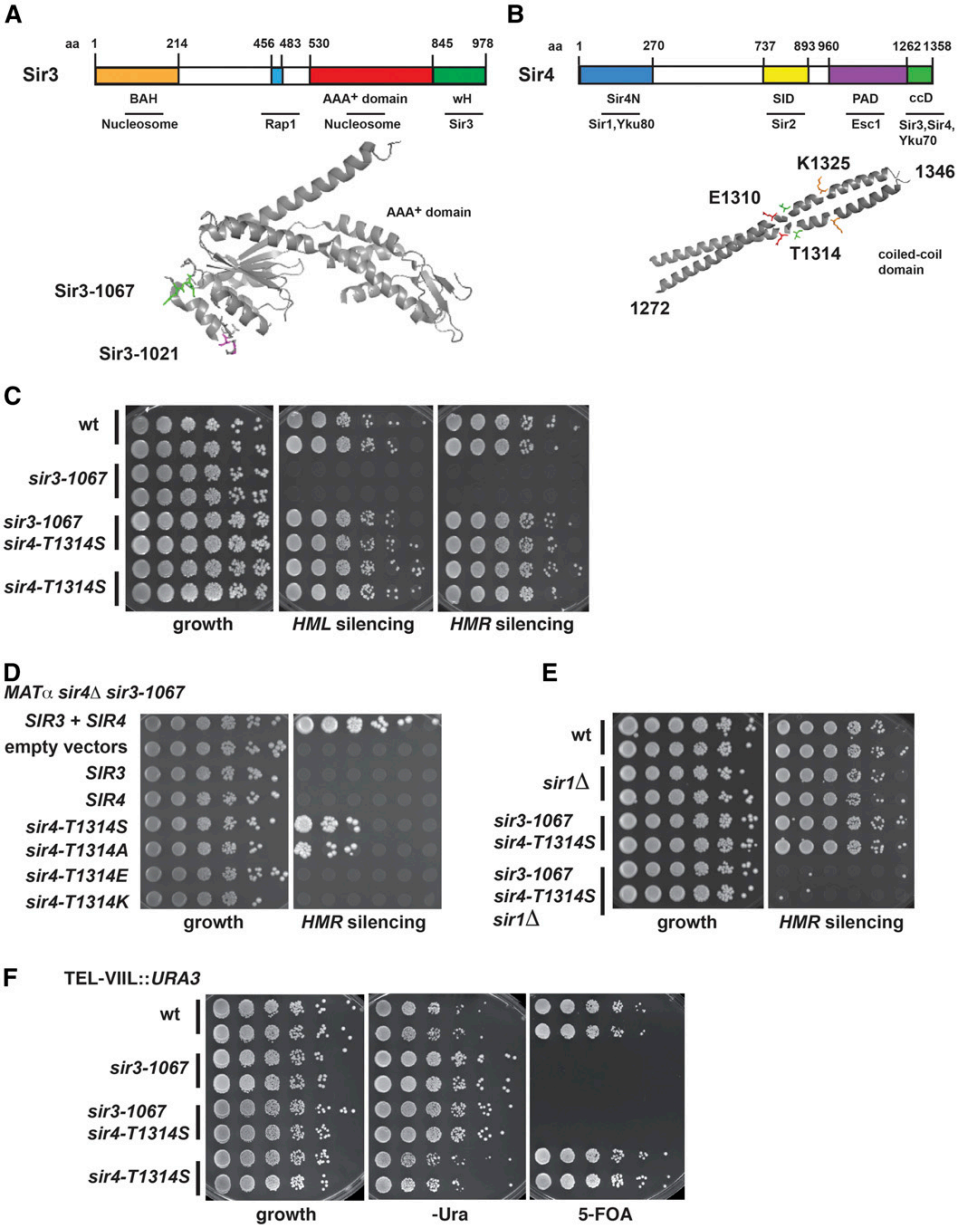
Identification of a mutation in the coiled-coil domain of Sir4 that suppresses the *HM* silencing defect of a mutation in the Sir3 AAA^+^ loop. (A) Schematic illustration of the Sir3 protein (top), and structure of the AAA^+^ ATPase-like domain (below). BAH, bromo-adjacent homology domain; wH, winged-helix domain. The *sir3* alleles *sir3-1067* (K657A, K658A) and *sir3-1021* (D640A, S642L) are mapped on the structure (PDB: 3TE6) (B) Schematic illustration of the Sir4 protein including the C-terminal coiled-coil domain (ccD, aa 1262–1358), the Sir2 interaction domain (SID), and the partitioning and anchoring domain (PAD) (top). Below, structure of the Sir4 coiled-coil domain. The amino acids E1310, T1314, and K1325 that are relevant for this study are mapped on the structure (PDB: 1PL5). (C) Mutation of Sir4-T1314 to serine (*sir4-T1314S*) restored the silencing defect of *sir3-1067* at *HMR* (AEY5554) and *HML* (AEY5555). A semiquantitative mating assay was performed as described in *Materials and Methods*, and plates were incubated for 3 d at 30°. (D) Mutation of Sir4-T1314 to serine and alanine (*sir4-T1314S*, *sir4-T1314A*), but not glutamine (E) or lysine (K), restored the *HMR*-silencing defect of *sir3-1067*. Plasmids encoding the respective Sir3 and Sir4 versions were transformed into a *MAT*α *sir3-1067 sir4Δ* strain (AEY5184). (E) Suppression of *sir3-1067* by *sir4-T1314S* depended on Sir1. (F) *Sir4-T1314S* did not suppress the telomeric silencing defect of *sir3-1067*. *sir3-1067* and *sir4-T1314S* were chromosomally integrated into a TEL-VIIL::*URA3* strain, and serial dilutions were spotted on a 5-FOA containing plate to analyze their ability to silence the *URA3* reporter gene at the telomere. Cells were additionally spotted on supplemented minimal medium as a growth control. The plates were incubated for 3 d at 30°.

The largest protein in the SIR complex is the Sir4 protein, which undergoes a multitude of protein–protein interactions to ensure efficient repression (Figure 1B), and thus is regarded as a scaffold protein for silencing (Kueng *et al.* 2013). Little structural information is available for Sir4, as its nonglobular nature has hindered its biochemical analysis. Apart from the above-mentioned dimerization with Sir2, Sir4 also interacts with the Ku heterodimer (Roy *et al.* 2004) as well as with Rap1 (Moretti *et al.* 1994; Kueng *et al.* 2012). The partitioning and anchoring domain of Sir4 (PAD, aa 960–1262) also mediates anchorage to the nuclear envelope via interaction with Esc1, which is associated with the nuclear envelope (Taddei *et al.* 2004). The N-terminus of Sir4 interacts with naked DNA and with Sir1 (Triolo and Sternglanz 1996; Martino *et al.* 2009; Kueng *et al.* 2012), which serves to recruit Sir2/Sir4 to the silencers. The only further structural information available on Sir4 is from the extreme C-terminus (aa 1272–1358), which dimerizes and assumes a parallel coiled-coil conformation (Chang *et al.* 2003; Murphy *et al.* 2003). Mutations that disrupt the dimerization activity abrogate Sir4 silencing function. Importantly, the Sir4 coiled-coil is sufficient to interact *in vitro* with a Sir3 fragment comprising the AAA^+^ domain and the wH domain (aa 464–978), and mutations were identified in a hydrophobic patch on the surface of the coiled-coil that do not interfere with dimerization, but abrogate the *in vitro* interaction with Sir3 (Chang *et al.* 2003). A previous study showed that these mutations also abrogated *in vivo* silencing (Rudner *et al.* 2005).

Altogether, the SIR complex contains two known dimerization domains, the Sir3 wH (Oppikofer *et al.* 2013b), and the Sir4 coiled-coil (Chang *et al.* 2003; Murphy *et al.* 2003). The SIR complex therefore possibly can form a heterohexamer with two subunits each of Sir2, Sir3, and Sir4. Each heterohexamer contains two Sir3 BAH, and two AAA^+^ domains, both of which bind nucleosomes, and whose binding is further supported by the Sir3 wH.

Here, we sought to characterize the defect of mutations in the interaction loop of the Sir3 AAA^+^ domain with Sir4 (Ehrentraut *et al.* 2011). We hypothesized that the Sir3 loop interacts directly with surface residues of the Sir4 C-terminus. Our work led us to identify a point mutation in the Sir4 coiled-coil domain, a mutation of threonine 1314 to serine (*sir4-T1314S*), that was able to suppress the silencing defect of the Sir3 loop mutation at *HMR* and *HML*. Interestingly, further mutations in the vicinity (E1310V, K1325R) were required to also restore telomeric silencing. We show that these mutations restore two-hybrid interactions with Sir3. Furthermore, the mutant Sir4 coiled-coil domains were capable of *in vitro* association with Sir3 versions with a defective Sir4 interaction loop. Altogether, our work shows the surprising discovery that removal of a single methyl group at position 1314 of Sir4 is sufficient to enhance binding between the Sir4 coiled-coil and Sir3, and thus to enhance heterochromatin formation *in vivo*.

## Material and Methods

### Yeast strains and plasmids

The yeast strains and plasmids used in this study are listed in Supplemental Material, Table S1 and Table S2. Yeast was grown and manipulated according to standard procedures (Sherman 1991). Yeast was grown on full medium (YPD) and selective minimal plates (YM), and plates containing 5-fluoro-orotic acid (US Biological) were used to select against *URA3*. Chromosomal integration of *sir4* alleles was obtained by transferring them onto a yeast integrating plasmid (pRS306, *URA3*-marked), and introducing them into yeast strains by integrative transformation followed by loop-out on 5-FOA medium. Semiquantitative mating assays were performed by generating serial dilutions (1:10, start OD600 of one) of the respective strain in a microtiter dish. For the growth control, cells were transferred to agar plates using a replica tool. An equal volume of the mating tester strain (suspension of 10 OD600 per milliliter) was then added to the strain in the microtiter well, and a replica of this mixture was transferred to a plate selective for the growth of diploids. Plates were incubated for 2–3 d at 30°.

pGBD-C2-*sir4* plasmids were generated by excising a *Cla*I/*Blp*I fragment of *sir4* alleles from pAE2289 or pAE2029, and inserting it into *Cla*I/*Blp*I-cleaved pAE1355. pGAD-C2 and pGBD-C2 plasmids encoding the coiled-coil domain of Sir4 were generated by inserting Sir4 fragments using *Cla*I and *Bgl*II.

### Random mutagenesis of SIR4

To isolate *sir4* alleles that suppress the *HMR* silencing defect of *sir3-1067*, a region of *SIR4* corresponding to aa 747–1358 was amplified from a *URA3-SIR4* plasmid (pAE233) using mutagenic PCR conditions (van Loo *et al.* 2004). The plasmid was cleaved with *Cla*I/*Sma*I, and the backbone was cotransformed with the mutagenized PCR product into a *MAT*α *sir3-1067 sir4*∆ strain (AEY5184) in order to generate *URA3-sir4* plasmids by gap repair. Resulting transformants were tested for their ability to mate with a *MAT*a tester strain. Among ∼15,000 transformants, one candidate was identified that restored mating ability. The *URA3-sir4* plasmid was isolated from the candidate, amplified in *Escherichia coli*, retested in AEY5184 for restoration of mating, and subjected to sequencing. The plasmid carried 17 mutations compared to *SIR4*, 10 of which caused aa changes. Further investigation showed that the mutation causing the suppression phenotype lies between aa 1142 and 1358 of the mutant *sir4* allele, which carries 2 aa changes (T1314S and V1351A). The *sir4-T1314S* mutation was constructed *de novo* by PCR sewing and gap repair to generate pAE2112. The equivalent procedure was carried out in a *MAT*α *sir3-1067 sir4*∆ TEL-VIIL::*URA3* strain (AEY5461), but using a *HIS3-SIR4* plasmid (pAE2137), and a total of 37,000 candidates were screened for restoration of silencing to the telomeric *URA3* reporter. This resulted in the identification of two candidate plasmids that restored telomeric silencing. One candidate carried nine mutations (four silent mutations) in *SIR4*, including E1310V and T1314S, and the other candidate had nine mutations (no silent mutations), including T1314S and K1325R.

### Expression and purification of recombinant Sir3 and Sir4 constructs

Sir3 (464–978 aa) constructs were cloned into pET21d using *Nco*I*/BamH*I. The constructs were expressed in *E. coli* strain *BL21 Rosetta*, and protein production was induced by auto-induction (Studier 2005). The purification was performed according to Chang *et al.* (2003) and King *et al.* (2006). Briefly, cell pellets were resuspended in lysis buffer containing 50 mM Hepes (pH 7.6), 500 mM KCl, 5% glycerol, 5 mM β-mercaptoethanol, 1 mM PMSF, protease inhibitors, and lysozyme. The lysate was incubated 30 min on ice. After sonification, the insoluble material was pelleted for 60 min at 20,000 × *g*. The lysate was incubated with Protino Ni-IDA resin (Macherey and Nagel) for 1 hr at 4°. Bound protein was washed and eluted with 250 mM imidazole. The eluate was subsequently diluted five times in 50 mM Hepes (pH 7.6), 1 mM EDTA, 1 mM PMSF, and 1 mM DTT, and eluted with a gradient of 100–500 mM KCl on a HiTrap FF Sepharose ion exchange column (GE Healthcare). Pooled fractions were concentrated, and loaded on a Superdex 200 gel filtration column. Peak fractions were pooled, concentrated, flash frozen in liquid nitrogen, and stored at −80°. Sir4 (1217–1358 aa) constructs were cloned into pET15b using *Xho*I/*BamH*I. Sir4 plasmids were transformed in *E. coli* strain *BL21 Rosetta*, and protein production was induced with 0.5 mM IPTG at 20° overnight. The cell pellets were resuspended in lysis buffer containing 50 mM NaH_2_PO_4_, 300 mM NaCl (pH 8), 1 mM PMSF, 1 mM β-mercaptoethanol, 0.2% Tween 20 and lysozyme. After lysis, the purification of Sir4 constructs with Ni-NTA agarose (Qiagen) was performed according to the manufacturer’s instructions. Subsequently, the Sir4 containing eluates were concentrated and loaded on a Superdex 75 gel filtration column. Pooled fractions were concentrated, flash frozen in liquid nitrogen, and stored at −80°.

### Analytical gel filtration

For analytical size exclusion chromatography (SEC) experiments, the proteins were mixed in equimolar ratio, and incubated for 40 min on ice. Subsequently, the SEC experiments were performed on a Superdex 200 Increase 3.2/300 column under isocratic flow conditions at 4°. The eluates were fractionated, loaded onto SDS-PAGE, and the gels were stained with Coomassie Brilliant Blue.

### RNA expression analysis

Total RNA from 50 ml yeast cultures was extracted with TriFast (Peqlab), followed by an additional DNase treatment and cDNA synthesis using SuperScript III reverse transcriptase (Invitrogen) according to the manufacturer’s protocol. Subsequently, quantitative real-time PCR was performed with Sybr Green Mastermix (Quanta) to determine the expression of the indicated subtelomeric genes. Primer sequences for quantitative real-time PCR are available upon request.

### Chromatin immunoprecipitation (ChIP)

ChIP and quantititative real-time PCR were performed as previously described with the following exceptions (Samel *et al.* 2012). For crosslinking, the cells were incubated for 20 min with 1% formaldehyde, and the reaction was stopped with 125 mM glycine for 5 min; 5 µl of α-His antibody (Sigma, H-1029) was used for the immunoprecipitation of Sir4. The cell lysate was subsequently incubated with Dynabeads Protein G (Invitrogen) for 5 hr at 4°. Primer sequences for quantitative real-time PCR are available upon request.

### Data availability

Strains and plasmids are available upon request. Table S1 contains the genotypes of all strains used in this study. Table S2 contains all plasmids used in this study.

## Results

### Removal of a methyl group by a threonine-to-serine mutation at Sir4 position T1314 restores HM silencing with Sir3 mutated in the AAA^+^ domain

Our earlier structure-function analysis of the Sir3 AAA^+^ domain showed that mutations in a loop of the AAA^+^ domain (K657A, K658A; named *sir3-1067*) abrogated Sir3 silencing function as well as the interaction of Sir3 with Sir4 [Figure 1A, and see Ehrentraut *et al.* (2011)]. Here, we sought to identify the region of Sir4, whose interaction with Sir3 is disrupted by *sir3-1067* by isolating alleles of *SIR4* that suppressed the silencing defect of *sir3-1067*. We reasoned that such mutant Sir4 proteins might restore silencing by improved binding to the mutant Sir3-1067 protein.

Since Sir3 has previously been shown to interact with the C-terminus of Sir4 (Chang *et al.* 2003; Murphy *et al.* 2003), we performed a random mutagenesis of the C-terminal region of a plasmid-encoded *SIR4* corresponding to aa 747–1358 (see *Materials and Methods* for details), and isolated suppressors of the *HMR* silencing defect of a *MAT*α *sir3-1067 sir4*Δ strain. Further analysis revealed that a single point mutation, threonine 1314 to serine (*sir4-T1314S*), was able to suppress the silencing defect of *sir3-1067* at *HMR* (Figure S1). Interestingly, this mutation is located within the coiled-coil domain of Sir4 that has previously been shown to interact with Sir3 [Figure 1B, and see Chang *et al.* (2003)]. Sir4-T1314S was fully functional for Sir4 function in cells containing wild-type Sir3, since it provided wild-type silencing in a *sir4*Δ strain (Figure 1C and Figure S2A), showing that the substitution of threonine to serine did not interfere with normal Sir4 function.

Since Sir4 protein levels can vary when expressed from a plasmid, which might influence silencing levels (Cockell *et al.* 1998; Larin *et al.* 2015), we determined the effect of *sir4-T1314S* when genomically encoded from the native *SIR4* locus. Sir4-T1314S was expressed at similar levels as the wild-type Sir4 protein as determined by Western blotting (Figure S2B). Importantly, genomically encoded *sir4-T1314S* restored silencing at *HMR* as well as *HML* to wild-type levels, as measured by the ability of *sir3-1067 sir4-T1314S* strains to form diploids with tester strains of opposite mating type (Figure 1C). This showed that the suppression was not due to abnormal levels of plasmid-encoded Sir4.

We next asked whether the suppression of *sir3-1067* depended on the chemical nature of the substitution at position 1314 of Sir4. Interestingly, as for Sir4-T1314S, the substitution of threonine to alanine (*sir4-T1314A*) was able to suppress the silencing defect of *sir3-1067* (Figure 1D). Conversely, mutations to lysine (K) or glutamic acid (E) showed no restoration of silencing at *HMR*, and these substitutions caused a loss of Sir4 function in a *sir4*Δ strain (Figure S2A). This indicated that a positively or negatively charged aa at position 1314 led to surface charge changes that interfere with Sir4 function, whereas a neutral substitution improved Sir4 silencing function, potentially by altering Sir4 interaction with Sir3.

Although *sir4-T1314S* supported *HM* silencing levels in *sir3-1067* that were comparable to those of the wild-type Sir4 and Sir3 proteins (Figure 1C), this silencing was exquisitely sensitive to the absence of Sir1 (Figure 1E), which is in stark contrast to the subtle silencing defects at *HMR* in *sir1*∆ cells that express wild-type Sir3 and Sir4 (Rine and Herskowitz 1987). Also, *sir4-T1314S* was unable to suppress the silencing defect of *sir3-1067* at telomeres as measured by silencing of *URA3* integrated at telomere VII-L (Figure 1F). This indicated that, while *sir4-T1314S* can suppress some aspects of the *sir3-1067* phenotype, it did not completely suppress *sir3-1067*, raising the question whether other amino acid changes in Sir4 might have a more penetrant phenotype, and restore silencing function at the telomeres.

### Restoration of telomeric silencing in AAA^+^-mutated Sir3 by amino acid changes E1310V, T1314S, and K1325R in Sir4

Since *sir4-T1314S* was unable to suppress the telomeric silencing defects of *sir3-1067*, we screened for stronger alleles of *SIR4* that restored telomeric silencing in *sir3-1067 sir4*∆ cells. Remarkably, even though we started the mutagenesis with wild-type *SIR4*, we isolated two *sir4* suppressor alleles that both contained, in addition to other amino acid changes, the mutation T1314S that we had previously isolated as a suppressor of the *HMR* silencing defect. One allele also contained the mutation E1310V, whereas the other allele additionally carried K1325R. We therefore created new alleles with either mutation alone, all possible double mutants, and the triple mutant, and tested their ability to suppress the silencing defects of *sir3-1067* at the telomeres, and at *HMR*. Importantly, none of these mutations alone supported telomeric silencing in *sir3-1067* cells, and only *sir4-T1314S* suppressed the *HMR* silencing defect (Figure S3). Although *sir4-E1310V*, *T1314S* and *sir4-T1314S*, *K1325R* partially suppressed telomeric silencing in *sir3-1067*, *sir4-E1310V*, *K1325R* (*i.e.*, without *T1314S*) did not. The strongest suppression at all loci was observed in the triple mutant *sir4-E1310V*, *T1314S*, *K1325R* (referred to as *sir4-ETK* below). Sir4-ETK function did not depend on the expression of the mutant Sir3-1067, as a plasmid-borne *sir4-ETK* fully suppressed *HMR* silencing defects in *sir4*∆ *SIR3* cells (Figure S4).

**Figure 2:**
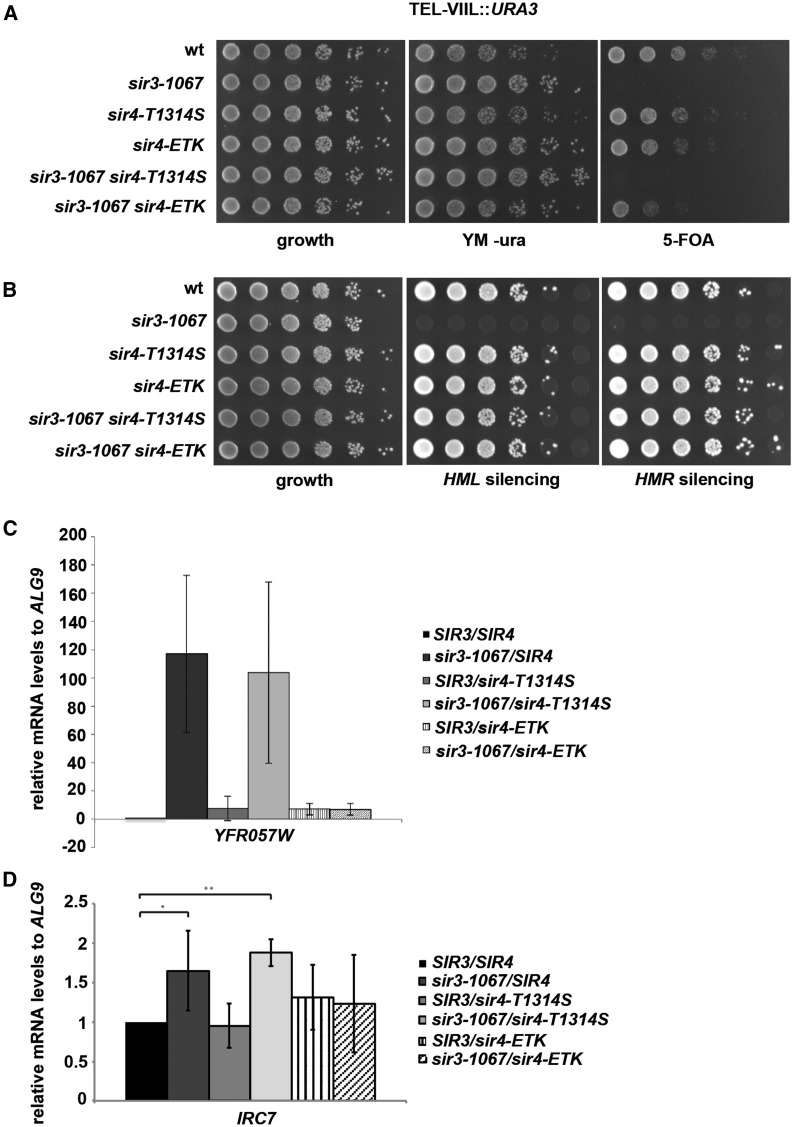
Sir4-E1310V, T1314S, K1325R restored *sir3-1067* silencing defects at the telomeres as well as the *HM* loci. (A) *Sir4-E1310V*, *T1314S*, *K1325R* (*sir4-ETK*) restored telomeric silencing in *sir3-1067*. The silencing assay was performed as in Figure 1F. (B) *Sir4-ETK* suppressed the silencing defect of *sir3-1067* at *HML* and *HMR*. Mating assays were performed as in Figure 1C. (C) *sir4-ETK* suppressed the expression of the subtelomeric gene YFR057W on chromosome VI-R in a *sir3-1067* strain background. Relative mRNA levels of the subtelomeric gene YFR057W relative to *ALG9* mRNA level was measured via qPCR. Errors bars give SDs of three biological triplicates. (D) *sir4-ETK* suppressed the expression of the subtelomeric gene IRC7 in a *sir3-1067* background. Representation as in Figure 2C. The asterisks indicate significant differences, * *P* < 0.05, ** *P* value < 0.005.

As above, to avoid potential complications of plasmid-encoded Sir4, we determined the silencing levels provided by genomically encoded *sir4-ETK*, which showed Sir4 protein levels that were comparable to wild-type Sir4 protein (Figure S2B). Significantly, this allele restored wild-type silencing levels to *sir3-1067* at *HMR* and *HML*, and nearly wild-type silencing of the telomeric *URA3* reporter (Figure 2, A and B).

We furthermore investigated the expression of native subtelomeric genes in the different mutant contexts. In agreement with the results from the telomeric *URA3* reporter, *sir3-1067* caused derepression of the subtelomeric genes *YFR057W* and *IRC7*, and this derepression was suppressed by *sir4-ETK*, but not *sir4-T1314S* (Figure 2, C and D).

We furthermore measured chromatin association of Sir4, Sir4-T1314S, and Sir4-ETK by ChIP. The levels of the mutant Sir4 versions were slightly reduced as compared to wild type at the telomeres as well as at the *HM* loci, even though the epitope-tagged versions used for ChIP were fully functional (Figure S5).

Altogether, these results suggest that the amino acid change T1314S improves interaction of Sir4 to a silencing interaction partner, possibly to Sir3, to restore *HM* silencing in *sir3-1067*, and that this interaction is further strengthened by the substitutions E1310V and K1325R.

**Figure 3:**
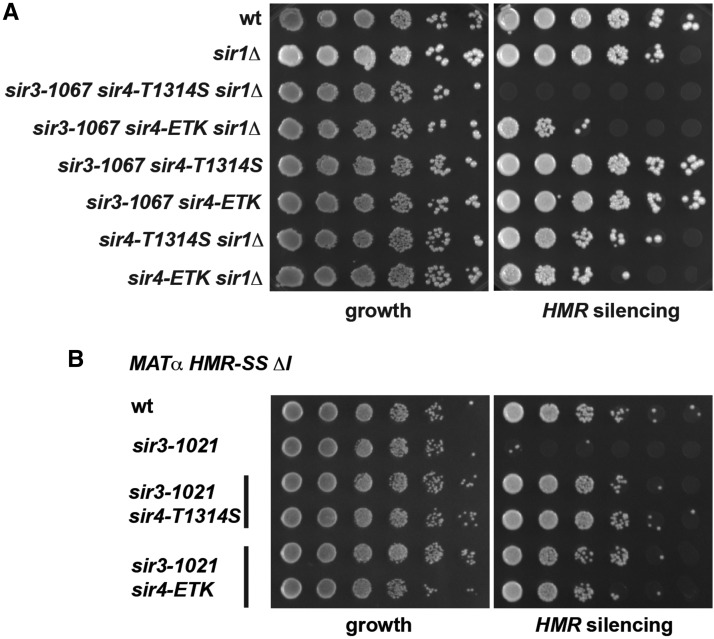
Suppression of the *sir3-1067 HMR* silencing defect by *sir4-T1314S* and *sir4-ETK* was impaired by deletion of *SIR1*. (A) Semiquantitative mating was performed as in Figure 1C. (B) *Sir4-T1314S* and *sir4-ETK* suppressed the silencing defect of *sir3-1021* at an *HMR* allele carrying a synthetic version of the *HMR* silencer and lacking *HMR*-I (*HMR-SS* ∆*I*). Mating assays were performed as above.

We furthermore asked whether the suppression of *sir3-1067* by *sir4-ETK* was dependent on the presence of Sir1. Interestingly, while there was a substantial loss of *HMR* silencing by *sir1*∆ in *sir3-1067 sir4-ETK* (Figure 3A), this loss was not as dramatic as that of *sir1*∆ in *sir3-1067 sir4-T1314S* (Figure 1E). Thus, *sir4-ETK* was more resilient toward perturbations than *sir4-T1314S*, which was in agreement with its ability to silence at telomeres. However, *sir4-ETK* (in the presence of wild-type *SIR3*) was more susceptible to *sir1*∆ than wild-type *SIR4* (Figure 3A), showing that this allele cannot fully replace the wild-type *SIR4*.

We furthermore asked whether *sir4-T1314S* and *sir4-ETK* were allele-specific suppressors of *sir3-1067*, or whether they were able to suppress another *sir3* allele. We therefore tested suppression of *sir3-1021*, which carries the mutations D640A and S642L (Figure 1A), which do not abrogate interaction with Sir4 (Ehrentraut *et al.* 2011), and which causes derepression at an *HMR* allele carrying a synthetic *HMR*-E silencer and lacking *HMR*-I (*HMR-SS* ∆*I*). Importantly, the *HMR* silencing defect of *sir3-1021* was strongly suppressed by *sir4-T1314S* and *sir4-ETK* (Figure 3B), showing that these *sir4* alleles were able to provide silencing in another *sir3* mutant background. However, these mutants were unable to suppress the silencing defects of *sir3*∆ cells (not shown), showing that a mutant form of Sir3 needed to be present for suppression.

### Mutations in the Sir4 coiled-coil restore its in vitro interaction with mutant Sir3

**Figure 4:**
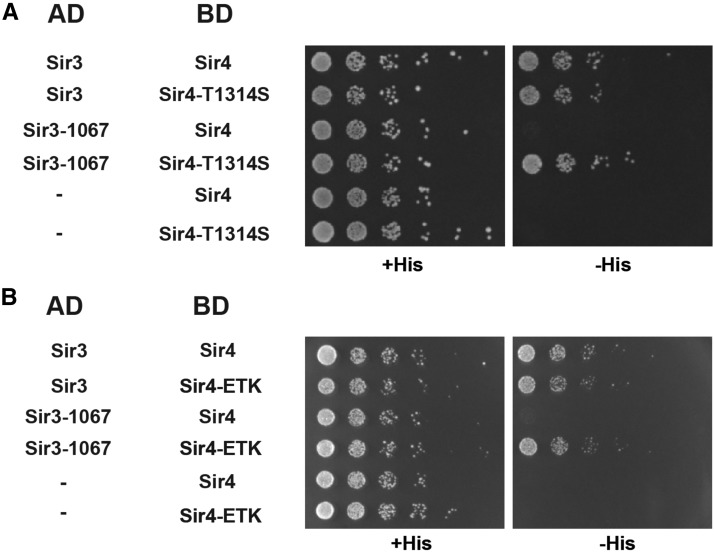
(A) Sir4-T1314S and (B) Sir4-E1310V, T1314S, K1325R (Sir4-ETK) restored the two-hybrid interaction to Sir3-1067. Strains [AEY3055 transformed with the respective plasmids carrying Sir3 (307–978) and Sir4 (839–1358)] were tested for activation of the two-hybrid reporter *HIS3* by plating serial dilutions on minimal medium with or without histidine.

We hypothesized that the amino acid changes identified here restored silencing function of Sir4 with mutant Sir3 because they restore the physical interaction between Sir4 and Sir3. Previous work has shown an interaction between the Sir4 coiled-coil and a C-terminal fragment of Sir3 (Chang *et al.* 2003), and we hypothesized that this interaction might be restored by the *sir4* mutants. As a first test of this hypothesis, we investigated the two-hybrid interactions of the different Sir3 and Sir4 mutant combinations. As reported earlier, Sir3 and Sir4 show a robust two-hybrid interaction, as measured by the growth of cells on medium lacking histidine as an indicator of activation of the *HIS3* two-hybrid reporter. Consistent with our previous work, Sir3-1067 was unable to interact with Sir4 (Ehrentraut *et al.* 2011). Strikingly, however, this interaction was restored by both Sir4-T1314S and Sir4-ETK (Figure 4), thus suggesting that the improved silencing could be attributed to improved binding between the mutant Sir3 and Sir4 proteins.

We also investigated other mutations in Sir4-T1314 for their two-hybrid interaction with wild-type and defective *sir3*. In agreement with the observation that *sir4-T1314A* suppressed *sir3-1067* silencing defects, this allele also restored its interaction with Sir3-1067 (Figure S6). In contrast, *sir4-T1314E* and *-K* were both unable to restore the interaction with mutant Sir3, which was in line with their inability to suppress *sir3-1067*. However, neither allele abrogated the interaction with wild-type Sir3, which was unexpected because they show a loss of Sir4 silencing function in *SIR3* cells, and thus are hypothesized to have lost interaction to Sir3. Thus, apparently, the *in vivo* silencing by Sir3 and Sir4 is more sensitive to perturbations than their two-hybrid interaction, and two-hybrid assay does not recapitulate all aspects of the physiologically relevant interactions of these proteins.

*sir4-T1314S* and *sir4-ETK* both mutate surface residues on the Sir4 coiled-coil, and we confirmed that these substitutions do not affect Sir4 dimerization as measured by a two-hybrid interaction. Neither mutant abrogated the interaction with wild-type Sir4 or to itself (Figure S7), indicating that they did not disrupt dimerization.

**Figure 5:**
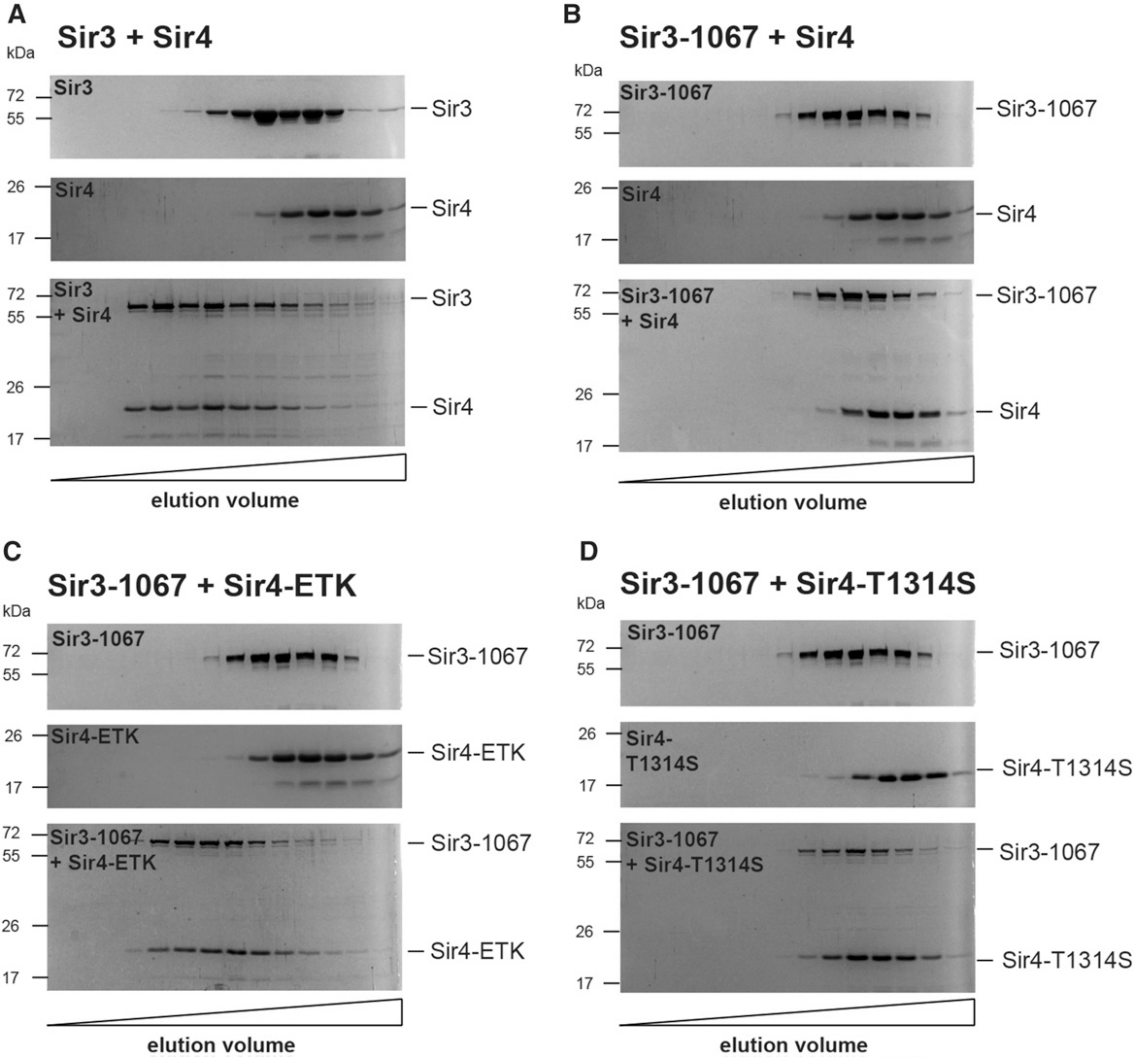
Sir4-ETK restored *in vitro* interaction to Sir3-1067. Sir4 (1217–1358) and Sir3 (464–978) as well as the respective mutant versions were expressed and purified separately from bacteria, and coincubated before separation by analytical gel filtration. (A) SDS-PAGE of SEC of wild-type Sir3 and Sir4, separately (top, middle), and after coincubation prior to SEC (bottom). Gels were stained with Coomassie Brilliant Blue. (B) SEC of Sir3-1067 with Sir4. Analysis is shown as in (A). (C) SEC of Sir3-1067 with Sir4-ETK. (D) SEC of Sir3-1067 with Sir4-T1314S.

In order to directly test whether the mutations in the Sir4 coiled-coil influenced the *in vitro* interaction of Sir4 with Sir3, we measured the *in vitro* interaction between bacterially produced and purified Sir3 (aa 464–978) and the Sir4 coiled-coil domain (aa 1217–1358) by size exclusion chromatography (SEC). Both fragments have previously been shown to behave roughly as dimers during gel filtration (Chang *et al.* 2003). Accordingly, in our SEC analysis, Sir3 (464–978) and Sir4 (1217–1358) alone eluted as single species. As expected, prior coincubation of the two wild-type proteins resulted in elution of both proteins at a higher apparent molecular mass, indicating Sir3–Sir4 complex formation (Figure 5A). Although the Sir3-1067(464–978) fragment showed similar behavior to the wild-type fragment when analyzed alone by SEC, coincubation with Sir4 (1217–1358) did not result in the formation of a higher-molecular weight complex (Figure 5B). This was in agreement with our earlier work that showed a loss of Sir3-1067 binding to full-length Sir2/Sir4 (Ehrentraut *et al.* 2011). Significantly, however, the Sir3-1067(464–978) fragment was capable of forming a high molecular weight complex with a Sir4-ETK (1217–1358) coiled-coil fragment (Figure 5C). This data showed that selected amino acid changes on the surface of the Sir4 coiled-coil increased *in vitro* binding to Sir3, and further indicated that the restoration of this interaction was the cause for improved silencing *in vivo*.

Although *sir4-T1314S* suppressed some silencing defects of *sir3-1067*, the purified Sir4-T1314S coiled-coil was not able to interact with the mutant Sir3 fragment (Figure 5D). This was surprising given that we had observed the restoration of the two-hybrid interaction between the two mutant proteins (Figure 4), and suggests that SEC may exaggerate defects in binding between these two proteins. Altogether, this showed that selected amino acid changes on the surface of the Sir4 coiled-coil increased *in vitro* binding to Sir3, and further indicated that the restoration of this interaction was the cause for improved silencing *in vivo*.

## Discussion

Multiple contacts among the Sir proteins are necessary for the assembly of a functional SIR complex that binds nucleosomes, and thus establishes heterochromatin. Here, we investigated the interaction between the Sir3 AAA^+^ domain and the Sir4 C-terminal coiled-coil. We made the surprising discovery that removal of a methyl group on the surface of the Sir4 coiled-coil domain (T1314S) restored silencing function in the presence of a Sir3 version with mutations in the Sir3 AAA^+^ domain. This site on Sir4 lies on the outer surface of the Sir4 coiled-coil [Figure 1B, and see Chang *et al.* (2003) and Murphy *et al.* (2003)]. We furthermore show that a combination of mutations on this surface restores the physical interaction between a mutant Sir3 AAA^+^ domain and Sir4, indicating that the restoration of this interaction is responsible for the regained silencing *in vivo*. We suggest that these mutations increase the affinity of Sir4 to Sir3.

Interestingly, surface residues on the Sir4 coiled-coil have previously been shown to be required for Sir3 interaction. Specifically, the changes M1307N, E1310R and I1311N in Sir4 caused a loss of *in vitro* interaction between Sir4 (1267–1358) and Sir3 (464–978) (Chang *et al.* 2003), and these three mutations disrupt the interaction between the full length proteins and silencing *in vivo* (Rudner *et al.* 2005). Remarkably, our mutation E1310V improves, rather than decreases, binding to Sir3, though only in conjunction with T1314S. It is also interesting to note that earlier studies found no effect on Sir4–Sir3 binding for the mutations Sir4-T1314N and K1325E (Chang *et al.* 2003; Rudner *et al.* 2005). We here identified other amino acid changes at these same sites that strengthened Sir4 binding to Sir3. Of note, K1325 lies within a hydrophobic surface of Sir4, via which two Sir4 coiled-coil dimers make crystal contacts (Murphy *et al.* 2003), and mutation of the nearby F1322 residue was shown to abrogate Sir4 silencing function. Our data suggest that this hydrophobic surface is, in fact, an interaction region with Sir3.

Based on the observation that mutations in Sir4 threonine 1314 abrogate the interaction with Sir3, one can speculate whether this residue might be regulated by post-translational modification, for instance phosphorylation. Such a modification is expected to disrupt the interaction to Sir3, since the phosphomimetic *sir4-T1314E* causes a silencing defect. However, phosphorylation at this site has not been observed so far (Kueng *et al.* 2012).

Where precisely is the contact of the Sir4 coiled-coil on Sir3? Our initial intention was to identify the site of contact with Sir4 of the loop in the Sir3 AAA^+^ domain by searching for site-specific suppressors. Although such mutations are expected to be allele-specific suppressors, we found suppression by the *sir4* alleles of a *sir3* mutation that lies one α-helix away from this loop (*sir3-1021*). This suggests that the Sir4 coiled-coil mutations increase binding to the Sir3 AAA^+^ domain, and that this enhanced binding may be sufficient to rescue other defects in Sir3. The contact therefore may be at the mentioned loop, but contact sites on other Sir3 surfaces are also possible. A further structural analysis will be required to resolve this question. We hypothesize that the Sir4 mutations increase binding not only to mutant, but also to wild-type Sir3. Interestingly, this may come at a cost for *HM* silencing, since *HMR* silencing by *sir4-ETK* was more sensitive to the loss of Sir1 function than silencing by wild-type *SIR4* (Figure 3A).

We also note with interest that there were differences in the “suppressability” of *sir3-1067* silencing defects at the *HM* loci compared to the telomeres, in that multiple mutations in the Sir4 surface were required to improve telomeric silencing. One interpretation of this observation is that a stronger interaction between Sir3 and Sir4 is required for telomeric silencing, because it is initiated by *cis* recruitment sites at the chromosome ends, and telomeric heterochromatin spreads unidirectionally toward subtelomeric regions. This is in contrast to *HM* silencing, where each silent locus is flanked on either side by an E and an I silencer as SIR recruitment sites, and SIR spreading proceeds in a convergent fashion. We suggest that the combined Sir4 mutations identified here increase the affinity of Sir4 to Sir3, and that this enhances SIR spreading, which is particularly important for telomeric silencing.

Another interpretation comes from the observation that the *sir4-ETK* is more proficient at *HMR* silencing in the absence of Sir1 than *sir4-T1314S*, which indicates that the increased affinity of Sir4-ETK to Sir3-1067 can overcome a nucleation defect at *HMR* caused by the absence of Sir1, whereas Sir4-T1314S cannot. Since telomeric silencing does not require Sir1 (Aparicio *et al.* 1991), the *sir3-1067* allele may require the stronger suppressor for telomeric silencing. The notion of increased affinity improving silencing is supported by earlier work showing that silencing of a derepressed *HM* silencer can be restored by perinuclear tethering to increased SIR protein concentration at the nuclear periphery (Andrulis *et al.* 1998; Taddei *et al.* 2009).

Interestingly, the restored *HMR* silencing achieved here was exquisitely sensitive to the recruitment factor Sir1, which binds to the *HM* silencers, and bridges the interaction of DNA binding proteins with the SIR complex. Sir1 has previously been shown to interact with the Sir4 N-terminus (Triolo and Sternglanz 1996; Kueng *et al.* 2012), *i.e.*, a region that, on the primary amino-acid sequence, is distant from the C-terminal coiled-coil. In this regard, it is interesting to note that the *sir4-ETK* mutant showed less *HMR* silencing in a *sir1*Δ mutant than the *sir4-ETK* mutant or *sir1*Δ mutant alone. One explanation is that the increased binding of *sir4-ETK* to wild-type Sir3 is detrimental for silencing in this context. How the Sir1 dependence reflects SIR binding and recruitment again will require more structural insights into the complex.

As in yeast, the formation of heterochromatin in higher eukaryotes relies on the spreading of chromatin-binding proteins like Heterochromatin Protein 1, or the Polycomb Repressive Complex 1. Learning about the basic principles of this process using the yeast SIR complex as a model will allow important insights into universal mechanisms of heritable chromatin silencing in eukaryotes.

## Supplementary Material

Supplemental material is available online at www.g3journal.org/lookup/suppl/doi:10.1534/g3.116.037739/-/DC1.
