# Supplementary material for: Variants of the Sir4 Coiled-Coil Domain Improve Binding to Sir3 for Heterochromatin Formation in Saccharomyces cerevisiae
Source: G3 (Bethesda). 2017 Feb 10;7(4):1117–26. doi: 10.1534/g3.116.037739 (PMC5386860; doi:10.1534/g3.116.037739)
Supplement: Supplementary file 1 [file 1117FigureS1.docx]

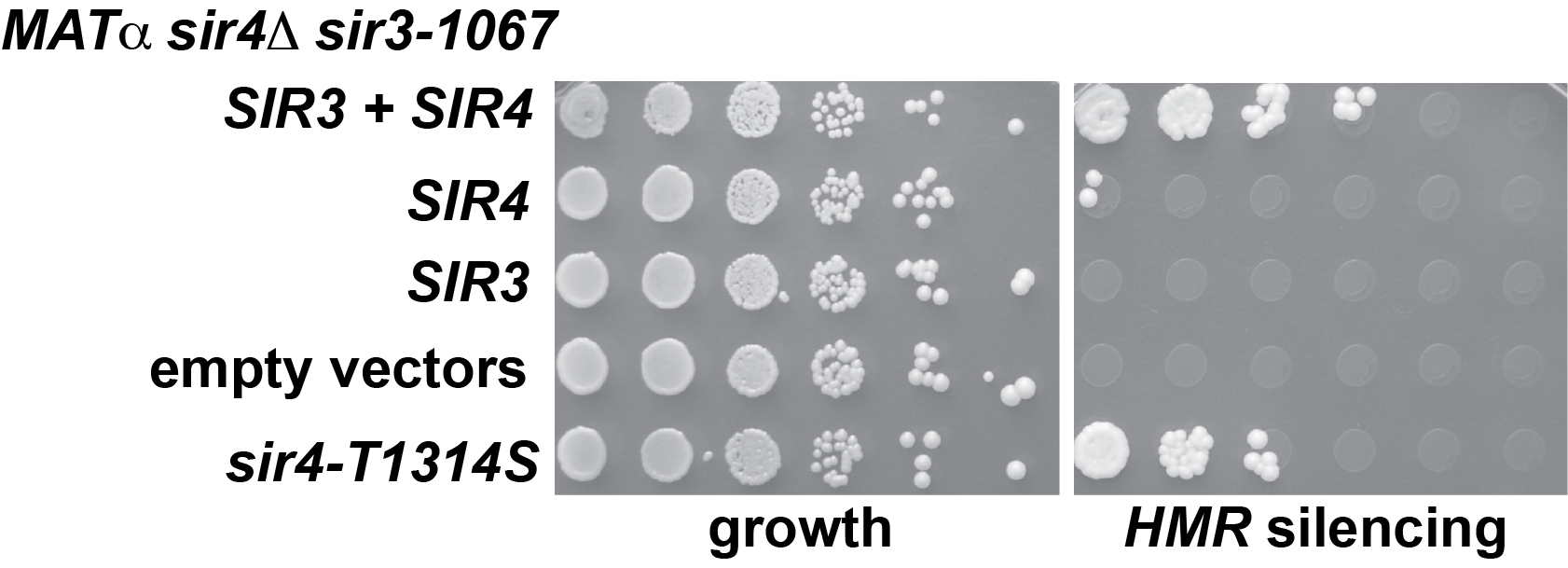


**Figure S1:**

The *HMR* silencing defect of *sir3-1067* was suppressed by plasmid-borne *sir4-T1314S*. Silencing assay was performed as described in Figure 1D.
