# Supplementary material for: Variants of the Sir4 Coiled-Coil Domain Improve Binding to Sir3 for Heterochromatin Formation in Saccharomyces cerevisiae
Source: G3 (Bethesda). 2017 Feb 10;7(4):1117–26. doi: 10.1534/g3.116.037739 (PMC5386860; doi:10.1534/g3.116.037739)
Supplement: Supplementary file 2 [file 1117FigureS2.docx]

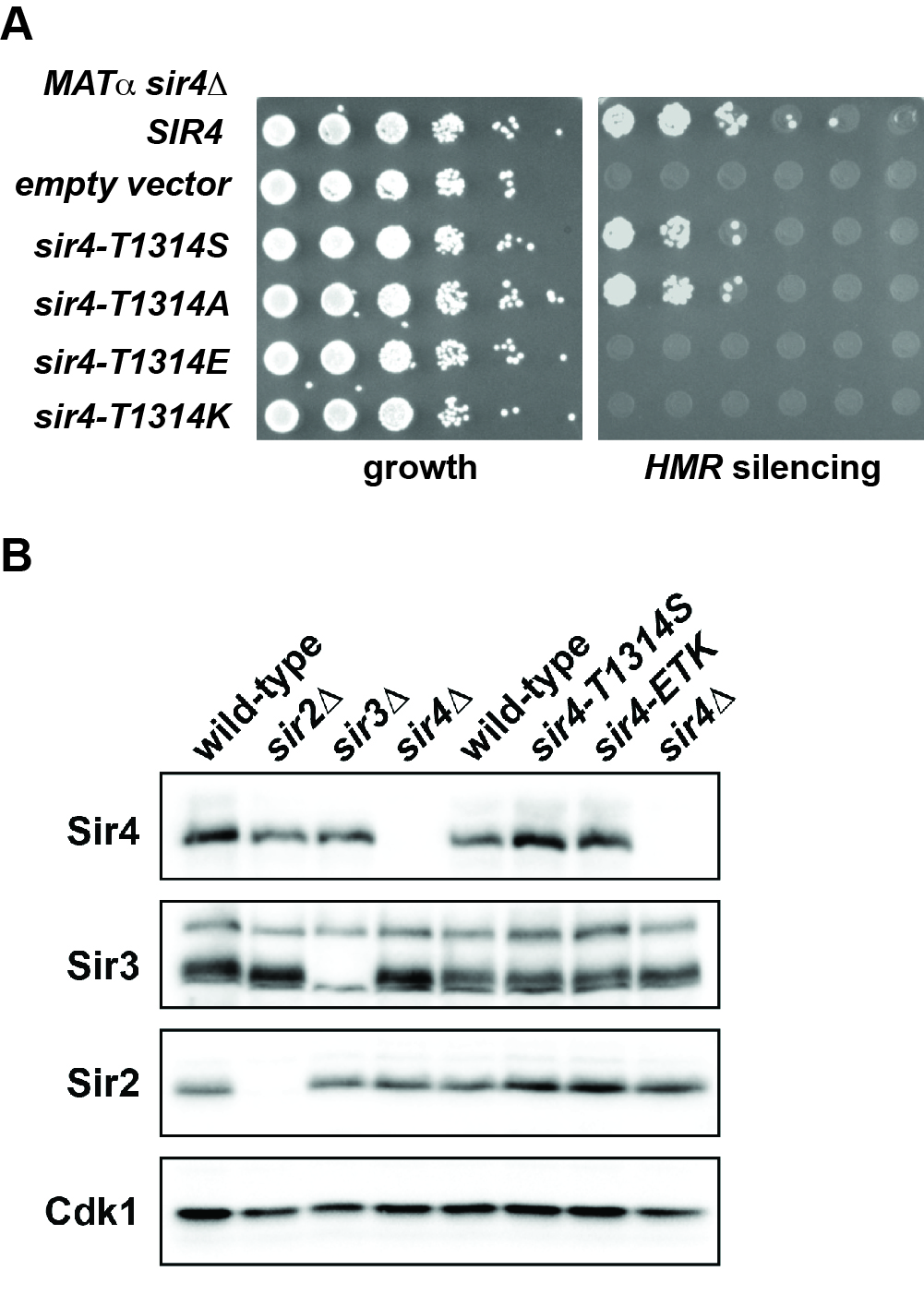


**Figure S2**:

(A) Plasmid-borne *sir4-T1314S* and *–A* showed wild-type levels of Sir4 function at *HMR*. The indicated plasmid-borne *sir4* alleles were introduced into a *MAT*α *sir4*Δ strain, and a semi-quantitative mating assay was performed as described in materials and methods. (B) Sir4-T1314S and Sir4-ETK protein levels were comparable to that of wild-type Sir4.
