# Supplementary material for: Variants of the Sir4 Coiled-Coil Domain Improve Binding to Sir3 for Heterochromatin Formation in Saccharomyces cerevisiae
Source: G3 (Bethesda). 2017 Feb 10;7(4):1117–26. doi: 10.1534/g3.116.037739 (PMC5386860; doi:10.1534/g3.116.037739)
Supplement: Supplementary file 3 [file 1117FigureS3.docx]

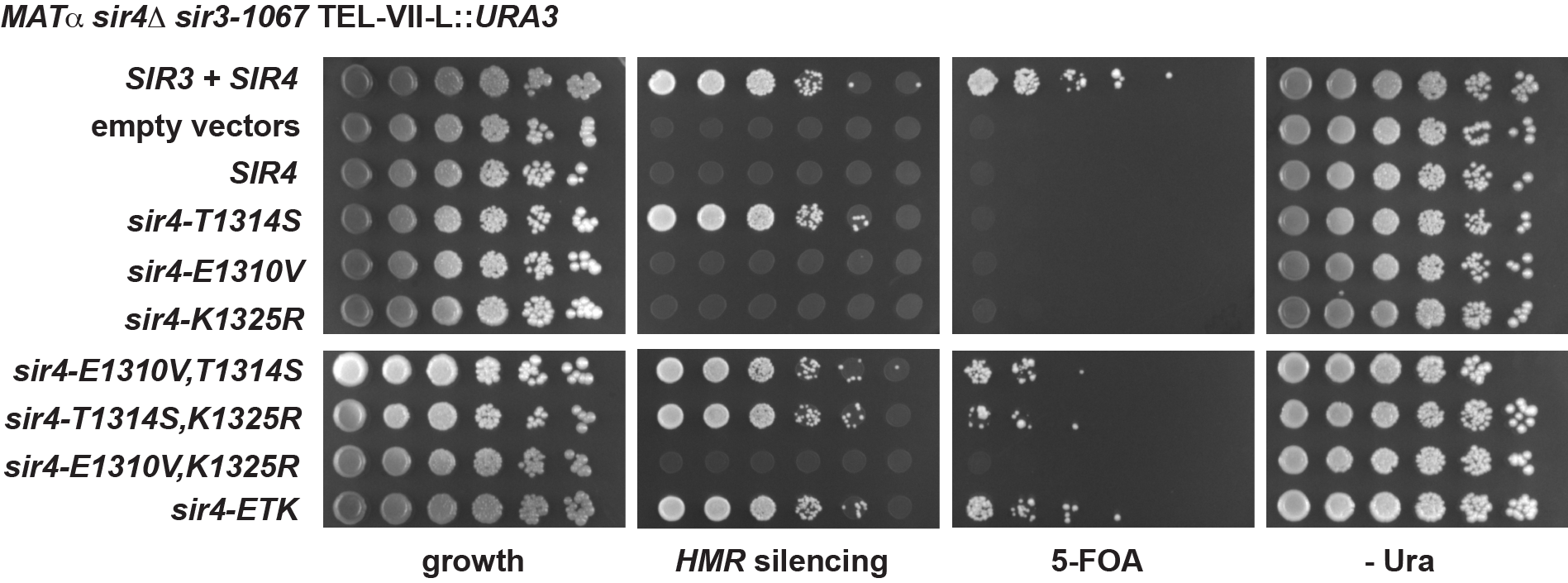


**Figure S3:**

Analysis of the ability of mutations in the *sir4* coiled-coil to suppress the silencing defect of *sir3-1067* at *HMR* and at the telomeres. Plasmid-borne *sir4* alleles were introduced into *MAT*α *sir4*Δ *sir3-1067* TEL-VIIL::*URA3,* and a semi-quantitative mating assay was performed as described in materials and methods. Additionally, the strains were plated on plates containing 5-fluoro-orotic acid for five days at 30 °C to test for their ability to silence the subtelomeric *URA3* reporter gene.
