# Supplementary material for: Variants of the Sir4 Coiled-Coil Domain Improve Binding to Sir3 for Heterochromatin Formation in Saccharomyces cerevisiae
Source: G3 (Bethesda). 2017 Feb 10;7(4):1117–26. doi: 10.1534/g3.116.037739 (PMC5386860; doi:10.1534/g3.116.037739)
Supplement: Supplementary file 4 [file 1117FigureS4.docx]

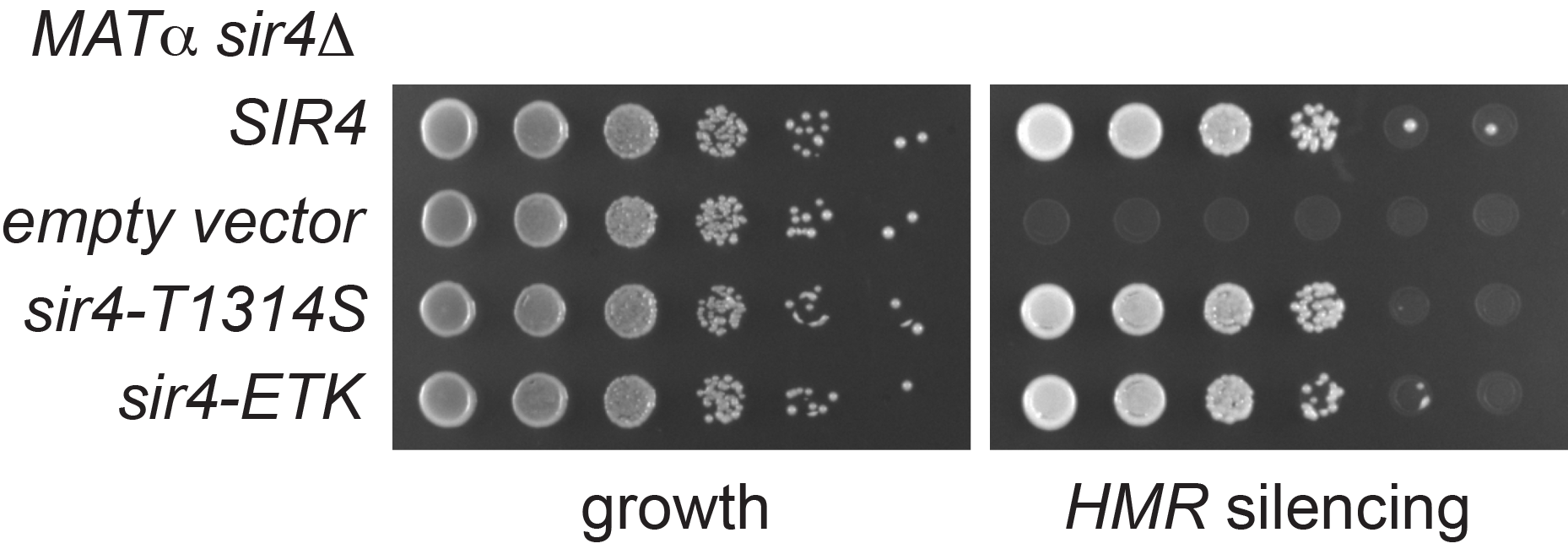


**Figure S4**:

*Sir4-T1314S* and *sir4-E1310V, T1314S, K1325R* (*sir4-ETK*) showed wild-type levels of Sir4 function at *HMR*. The indicated plasmid-borne *sir4* alleles were transformed into a *MAT*α *sir4*Δ strain, and a semi-quantitative mating assay was performed as described in materials and methods.
