# Supplementary material for: Variants of the Sir4 Coiled-Coil Domain Improve Binding to Sir3 for Heterochromatin Formation in Saccharomyces cerevisiae
Source: G3 (Bethesda). 2017 Feb 10;7(4):1117–26. doi: 10.1534/g3.116.037739 (PMC5386860; doi:10.1534/g3.116.037739)
Supplement: Supplementary file 5 [file 1117FigureS5.docx]

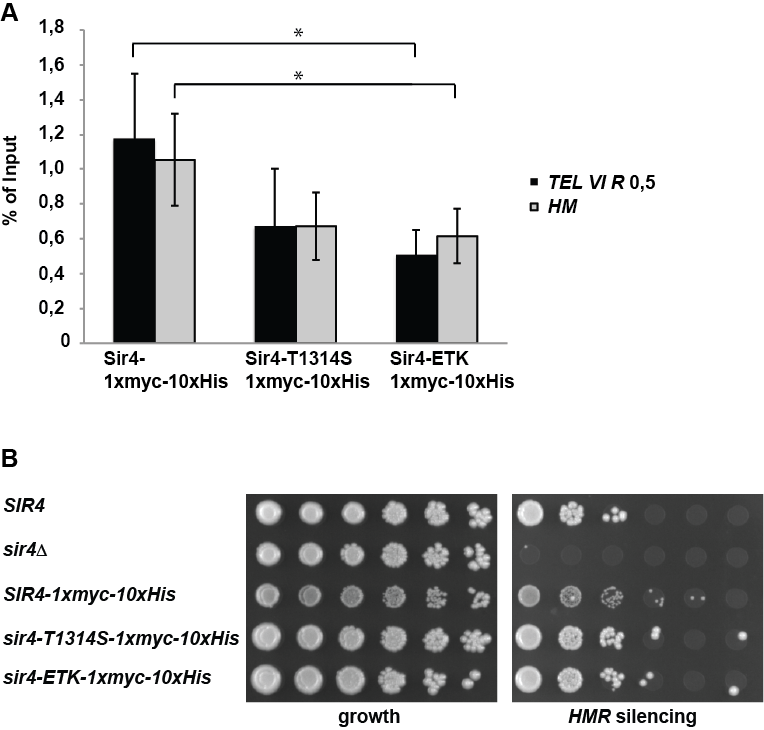


**Figure S5:**

Chromatin binding of Sir4-ETK was mildly reduced at *TELVI-R* and *HMR*. (A) The association of Sir4 and mutant versions at *TELVI-R* and *HMR* was measured by ChIP analysis. ChIP analysis was performed with α-His antibody as described in materials and methods. Values give the enrichment relative to input. Error bars give standard deviation of three independent ChIP experiments. The asterisk indicates significant difference, *P* value < 0,05. (B) C-terminal tagging of Sir4 and the Sir4 versions did not affect their function in *HMR* silencing. A semi-quantitative mating assay was performed as in Figure 1C.
