# Supplementary material for: Variants of the Sir4 Coiled-Coil Domain Improve Binding to Sir3 for Heterochromatin Formation in Saccharomyces cerevisiae
Source: G3 (Bethesda). 2017 Feb 10;7(4):1117–26. doi: 10.1534/g3.116.037739 (PMC5386860; doi:10.1534/g3.116.037739)
Supplement: Supplementary file 6 [file 1117FigureS6.docx]

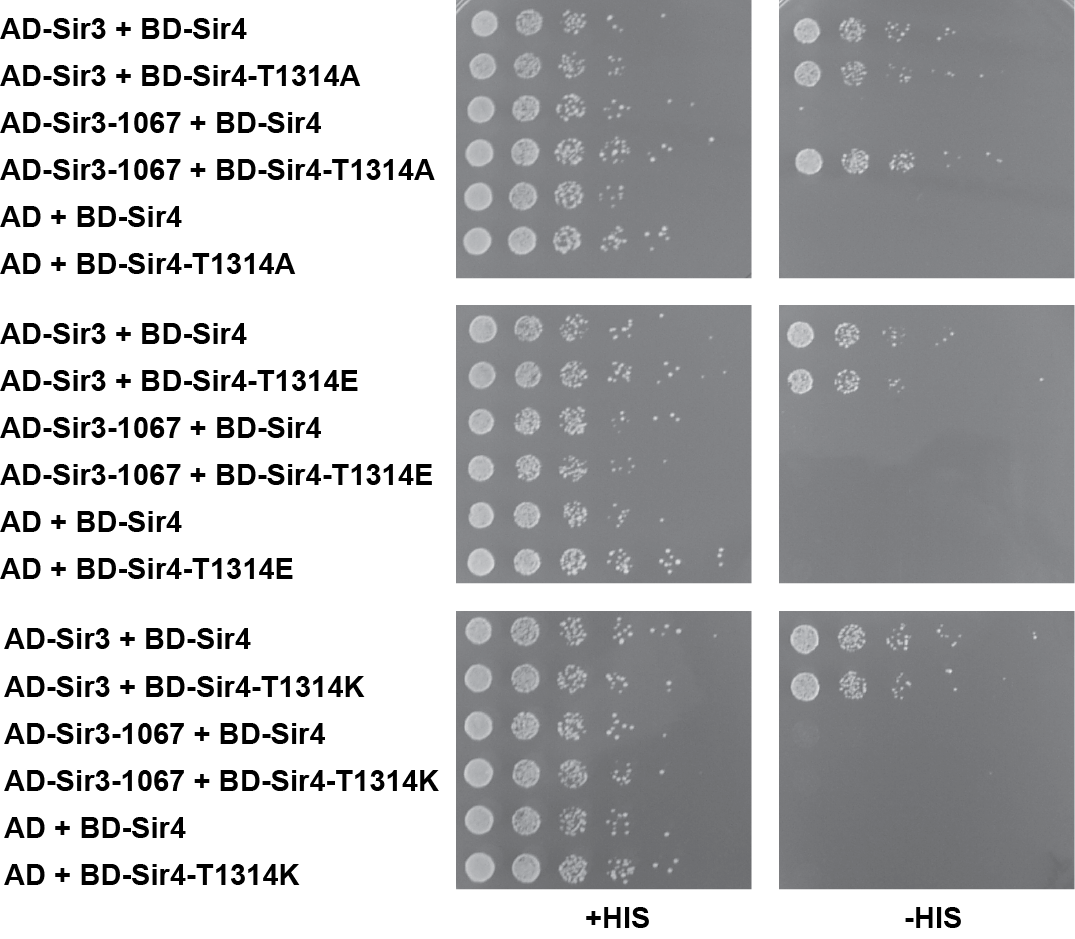


**Figure S6:**

Mutation of Sir4-T1314A restored interaction with Sir3-1067 in a two-hybrid assay. The indicated constructs of Sir3 (307-978aa) and Sir4 (839-1358aa) were transformed in a two-hybrid strain (AEY3055) and analysed for the ability to activate the *HIS3* reporter gene. Serial dilutions were spotted on minimal medium with and without histidine, and incubated 2 d at 30°C. Growing on medium without histidine indicated Sir3-Sir4 interaction.
