# Supplementary material for: Variants of the Sir4 Coiled-Coil Domain Improve Binding to Sir3 for Heterochromatin Formation in Saccharomyces cerevisiae
Source: G3 (Bethesda). 2017 Feb 10;7(4):1117–26. doi: 10.1534/g3.116.037739 (PMC5386860; doi:10.1534/g3.116.037739)
Supplement: Supplementary file 7 [file 1117FigureS7.docx]

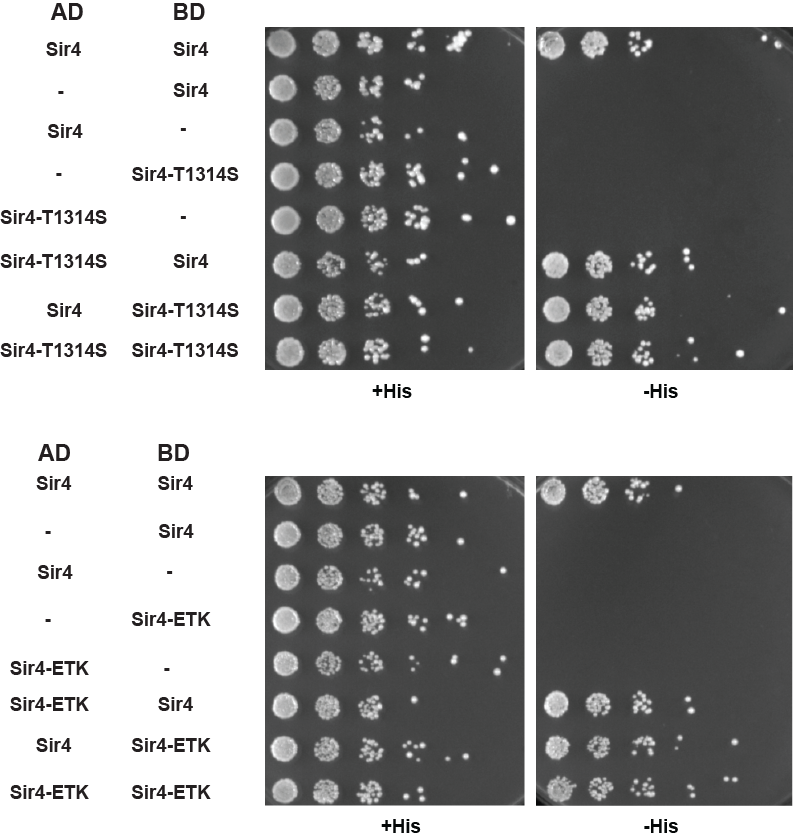


**Figure S7:**

Sir4-T1314S as well as Sir4-E1310V, T1314S, K1325R did not disrupt dimerization of the Sir4 coiled-coil domain. The indicated constructs of Sir4 (1262-1358aa) were transformed in a two-hybrid strain (AEY3055) and analysed for the ability to activate the *HIS3* reporter gene. Serial dilutions were spotted on minimal medium with and without histidine, and incubated 2 d at 30°C. Growing on medium without histidine indicated interaction of the coiled-coil domain of Sir4.
