# Supplementary material for: Variants of the Sir4 Coiled-Coil Domain Improve Binding to Sir3 for Heterochromatin Formation in Saccharomyces cerevisiae
Source: G3 (Bethesda). 2017 Feb 10;7(4):1117–26. doi: 10.1534/g3.116.037739 (PMC5386860; doi:10.1534/g3.116.037739)
Supplement: Supplementary file 8 [file 1117TableS1.docx]

**Table S1: Strains used in this study**

| Strain | Genotype | Source* |
| --- | --- | --- |
| AEY1 | *MATα ade2-101 his3-11,15 trp1-1 leu2-3,112 ura3-1* (W303) | J. Rine |
| AEY2 | AEY1, but *MAT***a** | J. Rine |
| AEY3 | AEY1, but *ADE2 lys2∆* | J. Rine |
| AEY4 | AEY2, but *ADE2* *lys2*Δ | J. Rine |
| AEY10 | *MATα sir1Δ::LEU2 ADE2* *lys2*Δ, W303 |  |
| AEY23 | *MATα sir4Δ::URA3 ADE2 lys2*Δ,  |  |
| AEY401 | *MATα HMR-SSΔI,*  |  |
| AEY3055 | *MAT***a** *trp1-901 leu2-3,112*  *ura3-52 his3-200 gal4Δ gal80Δ GAL2-ADE2 LYS2::GAL1-HIS3 met2::GAL7-lacZ* | P. James |
| AEY5181 | *MAT*α  *sir3-1067,*  |  |
| AEY5184 | *MAT*α  *sir3-1067 sir4Δ::KanMX,*  |  |
| AEY5461 | *MAT*α  *sir3-1067 sir4Δ::KanMX* TEL-VII-L*::URA3,*  |  |
| AEY5502 | *MAT*α *HMR-SSΔI sir3-1021 ADE2 lys2∆,*  |  |
| AEY5531 | *MAT*α *sir4-T1314S ADE2 lys2∆,*  |  |
| AEY5534 | *MAT***a** *sir3-1067 ade2 LYS2,*  |  |
| AEY5537 | *MAT***a** *sir3-1067 ADE2 lys2∆,* *+*pRS315*-SIR3* |  |
| AEY5554 | *MAT*α *sir3-1067 sir4-T1314S ADE2 lys2∆,*  |  |
| AEY5555 | *MAT***α** *sir3-1067 sir4-T1314S ADE2 lys2∆,*  |  |
| AEY5556 | *MAT***a** *sir4-T1314S ade2 LYS2,*  |  |
| AEY5560 | *MAT*α  *sir3-1067 sir4-T1314S ADE2 lys2∆* TEL-VII-L*::URA3,*  |  |
| AEY5567 | *MAT*α *sir4-T1314S ADE2 lys2∆* TEL-VII-L*::URA3,*  |  |
| AEY5582 | *MAT*α  *sir3-1067* TEL-VII-L*::URA3,*  |  |
| AEY5840 | *MAT*α  *HMR-SSΔI sir3-1021 sir4-T1314S ADE2 LYS2,*  |  |
| AEY5841 | *MAT*α  *HMR-SSΔI sir3-1021 sir4-T1314S ade2 lys2∆,*  |  |
| AEY5849 | *MAT*α *sir4-E1310V, T1314S, K1325R ADE2 lys2∆,*  |  |
| AEY5868 | *MAT*α *sir3*Δ::KanMX *sir4-E1310V, T1314S, K1325R ADE2 lys2∆,*  |  |
| AEY5881 | *MAT***a** *sir4-E1310V, T1314S, K1325R ade2 LYS2,*  |  |
| AEY5896 | *MAT*α *sir3-1067 sir4-E1310V, T1314S, K1325R ade2 LYS2,*  |  |
| AEY5888 | *MAT*α *sir3-1067 sir4-E1310V, T1314S, K1325R ade2 lys2Δ*, W303 |  |
| AEY5889 | *MAT***a** *sir3-1067 sir4-E1310V, T1314S, K1325R ADE2 LYS2,*  |  |
| AEY5897 | *MAT***a** *sir3*Δ::NatMX *sir4-E1310V, T1314S, K1325R ade2 LYS2,*  |  |
| AEY5899 | *MAT*α *sir3-1067 sir4-T1314S sir1Δ::KanMX ADE2 lys2Δ*, W303 |  |
| AEY5900 | *MAT*α *ade2 LYS2* TEL-VII-L*::URA3,*  |  |
| AEY5902 | *MAT*α *sir4-E1310V, T1314S, K1325R ADE2 lys2∆,*  |  |
| AEY5904 | *MAT*α  *sir3-1067 sir4-E1310V, T1314S, K1325R ade2 LYS2,*  |  |
| AEY5933 | *MAT*α *HMR-SSΔI sir3-1021 sir4-E1310V, T1314S, K1325R ade2 LYS2,*  |  |
| AEY5934 | *MAT*α *HMR-SSΔI sir3-1021 sir4-E1310V, T1314S, K1325R ade2 lys2∆,*  |  |
| AEY5938 | *MAT*α *sir4-T1314S sir1Δ::NatMX ADE2 lys2∆,*  |  |
| AEY5940 | *MAT*α *sir4-E1310V, T1314S, K1325R sir1Δ::NatMX ADE2 lys2∆,*  |  |
| AEY5942 | *MAT*α *sir3-1067 sir4-E1310V, T1314S, K1325R sir1Δ::NatMX ade2 lys2Δ*, W303 |  |
| AEY6082 | *MATα ADE2 lys2∆ SIR4*-1xmyc-10xHis::KanMX, W303 |  |
| AEY6083 | *MATα ADE2 lys2∆* *sir4-T1314S*-1xmyc-10xHis::KanMX, W303 |  |
| AEY6084 | *MATα ADE2 lys2∆* *sir4-ETK*-1xmyc-10xHis::KanMX, W303 |  |

*Unless indicated otherwise, strains were from the laboratory collection or were generated in the course of this study. All strains except AEY3055 are isogenic to W303.
