# Supplementary material for: Variants of the Sir4 Coiled-Coil Domain Improve Binding to Sir3 for Heterochromatin Formation in Saccharomyces cerevisiae
Source: G3 (Bethesda). 2017 Feb 10;7(4):1117–26. doi: 10.1534/g3.116.037739 (PMC5386860; doi:10.1534/g3.116.037739)
Supplement: Supplementary file 9 [file 1117TableS2.docx]

**Table S2:** **Plasmid used in this study**

| Plasmid | Description |
| --- | --- |
| pAE232 | pRS315*-SIR3* |
| pAE233 | pRS316-*SIR4* |
| pAE1301 | pGAD-C2 |
| pAE1304 | pGBD-C2 |
| pAE1352 | pGAD-C2-*SIR3* (307-978) |
| pAE1355 | pGBD-C2-*SIR4* (839-1358) |
| pAE1673 | pGAD-C2-*sir3-1067* (307-978) |
| pAE2029 | pRS316-*sir4-T1314S* |
| pAE2107 | pGBD-C2*-sir4-T1314S* (839-1358) |
| pAE2112 | pRS313-*sir4-T1314S* |
| pAE2137 | pRS313-*SIR4* |
| pAE2216 | pET15b-*SIR4* (1217-1358) |
| pAE2217 | pET15b-*Sir4-T1314S* (1217-1358) |
| pAE2262 | pGAD-C2-*SIR4* (1262-1358) |
| pAE2263 | pGAD-C2-*sir4-T1314S* (1262-1358) |
| pAE2264 | pGBD- C2-*SIR4* (1262-1358) |
| pAE2265 | pGBD-C2-*sir4-T1314S* (1262-1358) |
| pAE2276 | pRS313-*sir4-E1310V, T1314S* |
| pAE2277 | pRS313-*sir4-E1310V, T1314S, K1325R* |
| pAE2286 | pET21d-*SIR3* (464-978) |
| pAE2289 | pRS316-*sir4-E1310V, T1314S, K1325R* |
| pAE2290 | pRS313-*sir4-E1310V* |
| pAE2291 | pRS313-*sir4-K1325R* |
| pAE2292 | pRS313-*sir4-T1314S, K1325R* |
| pAE2293 | pRS313-*sir4-E1310V, K1325R* |
| pAE2307 | pGAD- C2-*sir4-E1310V, T1314S, K1325R* (1262-1358) |
| pAE2309 | pGBD- C2-*sir4-E1310V, T1314S, K1325R* (1262-1358) |
| pAE2326 | pGBD-C2-*sir4-E1310V, T1314S, K1325R* (839-1358) |
| pAE2357 | pET21d-*sir3-1067* (464-978) |
| pAE2359 | pET15b-*Sir4-E1310V, T1314S, K1325R* (1217-1358) |
